# Impaired functional cortical networks in the theta frequency band of patients with post-traumatic stress disorder during auditory-cognitive processing

**DOI:** 10.3389/fpsyt.2022.811766

**Published:** 2022-08-11

**Authors:** Miseon Shim, Han-Jeong Hwang, Seung-Hwan Lee

**Affiliations:** ^1^Industry Development Institute, Korea University, Sejong, South Korea; ^2^Department of Electronics and Information Engineering, Korea University, Sejong, South Korea; ^3^Interdisciplinary Graduate Program for Artificial Intelligence Smart Convergence Technology, Korea University, Sejong, South Korea; ^4^Psychiatry Department, Ilsan Paik Hospital, Inje University, Goyang, South Korea; ^5^Clinical Emotion and Cognition Research Laboratory, Goyang, South Korea

**Keywords:** post-traumatic stress disorder (PSTD), electroencephalogram, cognitive function, functional cortical networks, theta frequency band

## Abstract

Impaired cognitive function related to intrusive memories of traumatic experiences is the most noticeable characteristic of post-traumatic stress disorder (PTSD); nevertheless, the brain mechanism involved in the cognitive processing is still elusive. To improve the understanding of the neuropathology in PTSD patients, we investigated functional cortical networks that are based on graph theory, by using electroencephalogram (EEG). EEG signals, elicited by an auditory oddball paradigm, were recorded from 53 PTSD patients and 39 healthy controls (HCs). Source signals in 68 regions of interests were estimated using EEG data for each subject using minimum-norm estimation. Then, using source signals of each subject, time-frequency analysis was conducted, and a functional connectivity matrix was constructed using the imaginary part of coherence, which was used to evaluate three global-level (strength, clustering coefficient, and path length) and two nodal-level (strength and clustering coefficients) network indices in four frequency bands (theta, alpha, low-beta, and high-beta). The relationships between the network indices and symptoms were evaluated using Pearson’s correlation. Compared with HCs, PTSD patients showed significantly reduced spectral powers around P300 periods and significantly altered network indices (diminished strength and clustering coefficient, and prolonged path length) in theta frequency band. In addition, the nodal strengths and nodal clustering coefficients in theta band of PTSD patients were significantly reduced, compared with those of HCs, and the reduced nodal clustering coefficients in parieto-temporo-occipital regions had negative correlations with the symptom scores (Impact of Event Scale-Revises, Beck Depression Inventory, and Beck Anxiety Inventory). The characterization of this disrupted pattern improves the understanding of the neuropathophysiology underlying the impaired cognitive function in PTSD patients.

## Introduction

Patients with post-traumatic stress disorder (PTSD) generally suffer from recurrent recollection of distressing memories related to terrifying events (intrusive memory) and spontaneously tend to concentrate on negative thinking (rumination) biased toward traumatic events (1–5). Both these symptoms are distinctly presented only in PTSD patients. Due to the distinct symptoms of intrusive memory and rumination, PTSD patients show attention bias toward the traumatic events. Consequently, these patients develop cognitive impairments (4); they show lower performance in cognitive tasks, such as working memory, than healthy controls (HCs) (1, 5). Moreover, the impaired cognitive function in PTSD patients has been observed in objective physiological traits investigated using various functional neuroimaging modalities (2, 3, 6, 7).

Functional magnetic resonance imaging (fMRI) studies have investigated the altered cognitive function in PTSD patients, especially focusing on two regions of interests (ROIs), namely emotion-related areas (e.g., amygdala, insula, ventral anterior cingulate (ACC), and rostral ACC) and cognition-related areas (e.g., hippocampus and prefrontal regions, including dorsal ACC) (6, 8–10). In a previous study, when performing cognitive tasks *via* neutral stimuli excluding emotional information, the HCs showed significantly enhanced brain activation in cognition-related areas (11), whereas emotion-related areas were not significantly activated (12), meaning that the HCs appropriately allocated neuronal resources solely to cognition-related areas to process the cognitive tasks. However, PTSD patients display significantly enhanced activation in both emotion- and cognition-related areas, unlike healthy individuals (8), and the increased brain activation in emotion-related areas has a positive correlation with the hyper-arousal symptom of PTSD patients (8). These results indicate that PTSD patients allocate neuronal resources to not only cognition-related areas but also emotion-related areas even if emotional information is not involved in the cognitive tasks. In other words, in PTSD patients, neuronal resources that should be solely allocated to cognition-related areas are partially distributed to emotion-related areas when processing cognition-related information, leading to cognitive impairments.

Although previous fMRI studies have discovered the distinct regional information related to the impaired cognitive function in PTSD patients, it is not sufficient to fully understand the neuropathological characteristics, which has a resolution of several seconds, using fMRI because the cognitive function is processed very fast, within milliseconds (13, 14). To investigate the fast cognition-related neuronal processing, electroencephalogram (EEG) has been widely used due to its high temporal resolution (15), and the altered cognitive function in PTSD patients was revealed *via* EEG-based neurophysiological markers in terms of functional aspects (16). In particular, PTSD patients show a reduced amplitude of P300 event-related potential (ERP) and prolonged P300 latency than healthy individuals when performing cognitive oddball tasks, indicating that PTSD patients cannot allocate the appropriate amount of brain resources when processing cognition-related tasks (17, 18). Moreover, disrupted P300 characteristics have negative relationships with the psychiatric symptoms, such as avoidance and numbing, in PTSD patients (17). Thus, the distinct psychiatric symptoms of PTSD presumably lead to the cognitive decline (17). However, since various brain regions interact with each other to process cognitive information, simple ERP-based traits, such as peak amplitudes and latencies, are insufficient to comprehend the complex brain processing associated with cognitive function in PTSD patients (19). Therefore, brain network indices based on functional connectomes can be an appropriate alternative to improve the understanding of the neuronal mechanisms underlying complicated cognitive functions.

Indeed, several previous studies have shown the possibility of using brain network analysis based on graph theory to investigate the altered cognitive functions in other mental disorders (20–22). For example, when utilizing cognitive functions, schizophrenia patient’s show altered functional networks relative to those of healthy individuals, and the disrupted brain network traits in these patients are significantly correlated with the psychotic symptoms (20). Despite the promising results derived from the EEG-based network analyses, to the best of our knowledge, network analysis has not yet been applied to the EEG data from PTSD patients to investigate the dynamic disease-specific cognitive neural information.

In this study, we investigated the cortical network characteristics of PTSD patients by using EEG data measured during a simple cognitive task based on neutral auditory stimulation, to extend the understanding of PTSD neurophysiopathology in terms of the brain network. To this end, we computed weighted network indices (strength, clustering coefficient, and path length) by using cortical-level time series. Moreover, we explored the relationships between the network indices and clinical symptoms, thereby contributing to the understanding of the cognition-related neuropathology of PTSD.

## Materials and methods

### Participants

Fifty-three PTSD patients and 39 HCs were recruited for this study from the Psychiatry Department of Inje University Ilsan Paik Hospital. The patients had been diagnosed by a board-certified psychiatrist according to the Diagnostic and Statistical Manual of Mental Disorders, 4th edition (DSM-IV) Axis I Psychiatric Disorders. Patients were excluded if they had any of the followings: (1) abnormality of the central nervous system, (2) medical history of alcohol or drug abuse, (3) intellectual disability, (4) history of a head injury that resulted in loss of consciousness and experience of electrical therapy (e.g., electroconvulsive therapy), and (5) history of a psychotic symptom lasting for ≥24 h. For HCs, individuals without any psychiatric medical history were recruited from the local community through advertisements in local newspapers and posters. If the HCs had taken any psychotropic medication and, they were excluded from the study. This study protocol was approved by the Institutional Review Board of Inje University Ilsan Paik Hospital (2015-07-025) and conducted in accordance with The Code of Ethics of the World Medical Association (Declaration of Helsinki), and all the participants provided written informed consent.

### Evaluation of clinical symptoms

Two Psychometric tests were performed to check the psychiatric severity of the PTSD patients and HCs as well as to investigate the relationship between the psychiatric severity and cortical network indices. Beck Anxiety Inventory (BAI) (23) was used to evaluate the anxiety symptom (mild, 8–15; moderate, 16–25; and severe, 26–63) (24). Beck Depression Inventory (BDI) (25) was used to evaluate the depressive symptom (mild, 14–19; moderate: 20–28; and severe: 29–63) (26). These two psychiatric symptoms were evaluated for both PTSD patients and HCs. Additionally, Impact of Event Scale-Revises (IES-R) (27) was used to evaluate the responses to the traumatic events experienced by the PTSD patients (mild, 0–24; moderate, 25–39; severe, 40–59; and very severe, ≥ 60) (28, 29). The PTSD patients experienced the following traumatic events: severe motor-vehicle accidents (*n* = 46, 86.02%), physical and sexual abuse (*n* = 2, 3.77%), and other accidents, such as being inside a collapsing building and being trapped in an elevator (*n* = 5, 9.43%). The demographic data and psychiatric severity scores (BAI, BDI, and IES-R) of each group are reported in Table 1.

**TABLE 1:** Demographic data of the post-traumatic stress disorder (PTSD) patients and healthy controls (HCs).

	PTSD	HCs	*p*-value
Cases (N)	53	39	
Gender (male/female)	24/29	18/21	0.521
Age (years)	42.86 ± 10.49	38.74 ± 9.05	0.777
Education (years)	13.27 ± 3.13	14.47 ± 2.15	0.413
IES-R	53.21 ± 20.80		
BDI	26.58 ± 12.62	9.30 ± 5.13	0.001**
BAI	29.86 ± 15.71	5.13 ± 4.21	0.001**

The *p*-values represent significant differences between the two groups.

The *p*-values were obtained using the independent *t*-test for age and education, and the chi-squared test for gender.

BDI, back’s depression inventory; BAI, back’s anxiety inventory.

**p < 0.001.

### Experimental paradigm

The stimuli used for the auditory oddball paradigm were composed of target and standard tones with the tone frequencies of 1,500 and 1,000 Hz, respectively. The duration of each stimulus was set to 100 ms, and the rising and falling times were equally set to 10 ms. Four-hundred pure tone stimuli consisting of 15% target tones and 85% standard tones were presented in a random order with an inter-stimulus interval of 1,500 ms. The participants were required to press a response button whenever target tones were presented to check their attention to the target auditory stimuli.

### Electroencephalogram recordings and preprocessing

Electroencephalogram data were recorded with a 1–100 Hz band-pass filter at a sampling rate of 1,000 Hz from 64 Ag/AgCl scalp electrodes evenly mounted according to the extended international 10–20 system [NeuroScan SynAmps2 (Compumedics USA, El Paso, TX, United States); references, both mastoids]. Since mastoid-based references may cause distorted and spurious connections due to unwanted artifacts introduced outside of the brain when computing functional connectivity, re-referencing was conducted using common average reference (CAR). Eye-movement artifacts were removed using established mathematical procedures (30) based on the regression approach implemented in NeuroScan preprocessing software, and other gross artifacts were eliminated *via* visual inspection. Artifact-free EEG data were bandpass filtered using third Butterworth IIF filter with a forward-backward zero phase filtering process between 1 and 55 Hz and epoched between −1 and 1 s, based on the stimuli onset, and only target epochs were used for further analysis. The epochs were rejected if they contained significant physiological artifacts (± 100 μV) at any electrode (31). After performing the preprocessing steps, the number of remaining epochs were 59 ± 2.08 for PTSD patients and 59.05 ± 2.70 for HCs (*p* = 0.918), respectively, among all 60 target epochs. All other preprocessing steps, except EOG removal, were conducted using Matlab 2019b (MathWorks).

### Source estimation and time-frequency analysis

A lead-field matrix was computed using a three-layer (inner skull, outer skull, and scalp) boundary element method (BEM) model which was constructed from the standard head model (Colin 27) by using the Open MEEG toolbox (32). Note that to estimate accurate source signals, brain areas on cerebral cortex were only used to construct BEM model, excluding deep brain areas such as hippocampus and amygdala. An inverse operator was created using a weighted minimum-norm estimation (wMNE) algorithm implemented in the Brainstorm toolbox (33). A time series of source activities at 15,000 cortical vertices was estimated for each epoch. After computing the source-level time-series at each vertex, the representative source signals at 68 ROIs based on Desikan-Kiliany atlas were estimated using principal component analysis (34). The name of 68 ROIs is summarized in Supplementary Table 1. After computing source signals, time frequency analysis were performed to investigate whether accurate source signals were estimated, for which Fast Fourier Transform (FFT) with a single Hanning taper (three cycles long with respect to frequency bins) was performed for each trial (−200 to 1,000 ms based on the stimuli onset) to compute spectral powers. The power spectrum of each trial was then baseline normalized by subtracting the average power of the baseline period (−200 to 0 ms) from spectral powers of the task period (0 to 1,000 ms) and then the differences were divided by the average power of the baseline period to probe for changes in spectral powers before and after stimulus onset. The baseline normalized power spectra were then averaged over all trials, which resulted in baseline-normalized total power. Since the data length (1 s for task period) is too short to sufficiently cover the oscillatory activities in delta band, this band was excluded from further analysis (35, 36). Therefore, both total power and network analysis were conducted for four frequency bands [theta (4–8 Hz), alpha (8–12 Hz), low-beta (12–22 Hz), and high-beta (22–30 Hz)], respectively.

### Functional network analysis

Functional connectivity analysis was performed for network analysis; the imaginary part of coherence (iCoh), which implemented in FieldTrip toolbox, was computed using a time-series of the cortical activities at each ROI. iCoh was quantified based on spectral powers obtained using FFT with a single Hanning taper (three cycles long with respect to frequency bins). An iCoh matrix was evaluated between all possible pairs of 68 ROIs and quantified for four frequency bands. An iCoh matrix was independently computed for two time periods, as follows: baseline (−1 to 0 s) and task period (0 to 1 s), and the task-based iCohs were normalized by subtracting the baseline iCohs to extract the network indices directly related to the cognitive task. After the normalization processing, two different adjacency matrices were quantified as task-specific enhanced and diminished connectomes, indicating increased (positive) and decreased (negative) connectivity’s when performing the cognitive task, respectively, compared with the baseline period. Because use of both positive and negative connectomes can improve understanding of neural mechanisms related to cognitive processing for a complementary way, both positive and negative connectomes were independently analyzed, respectively. In this study, an original adjacency matrix was used to quantify network indices without any threshold to minimize information loss between all possible ROI pairs.

Two different types of weighted brain network indices were calculated based on graph theory for each of the positive and negative adjacency matrices. The first type consisted of the following three global-level network indices that take into account the whole-brain cortex: (1) strength (the degree of connection strength in the network); (2) clustering coefficient (the degree of functional segregation in the network, meaning the clustering degree between neighboring nodes); and (3) path length (PL, the degree of functional integration in the network, representing the overall connectedness of the whole network) (37). The second type consisted of two nodal (ROI)-level network indices that focus on the characteristics of individual brain regions—(1) strength and (2) clustering coefficient. All the network indices were computed using Brain connectivity Toolbox (BCT^1^) based on Matlab (38).

### Statistical analysis

Statistical tests were performed to investigate the significance of the difference between two groups for the total power and network analysis results independently. For multidimensional data (e.g., spectral power–4 ROIs × 1,201 time points × 27 frequency bins; nodal-level network indices–68 ROIs × 4 frequency bands), a cluster-based permutation test, implemented in Fieldtrip, was performed for correction of multiple comparisons (39). To this end, an independent *t*-test was conducted for each data point (e.g., spectral powers–for each ROI and each time and each frequency point), and clusters were identified based on two or more adjacent data points corresponding to *t*-values with a *p*-value less than 0.05. Then, a maximum *t*-value of each cluster was calculated by summing of all *t*-values within each cluster, and significant clusters were estimated by Monte Carlo method with 1,000 random permutations (*p* < 0.025, two-tailed). For the single-dimensional data, global-level network indices, a critical alpha level of 0.01 was set to correct the multiple comparison issue caused by four frequency bands, which is less than Bonferroni corrected alpha value (0.05/4 frequency bands = 0.0125). In addition, to investigate the relationships between the network indices of both global-and nodal-level network values and psychiatric-symptom scores of the PTSD patients and HCs, respectively, the Pearson’s correlation method was used with a permutation of 10,000 iterations (40, 41).

## Results

### Total power patterns

**FIGURE 1:**
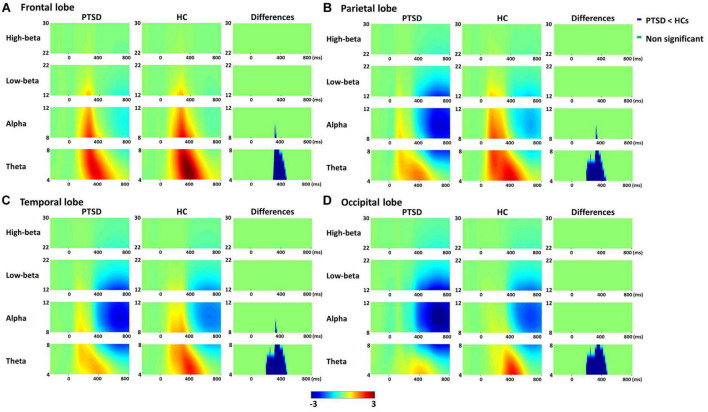
Averaged total power patterns of four lobes [frontal **(A)**, parietal **(B)**, temporal **(C)**, and occipital lobes **(D)**] across all subjects and region of interests (ROIs) included in each lobe for post-traumatic stress disorder (PTSD) patients and healthy controls (HCs), respectively, and the total power differences between PTSD patients and HCs.

Figure 1 represents total power pattern maps quantified for four different brain lobes (frontal, parietal, temporal, and occipital) by averaging all subjects of each group and ROIs included in each lobe for each frequency band. PTSD patients showed the significantly reduced cortical spectral powers in theta frequency band around 300 ms for all four lobes as compared to those of HCs (cluster-*p* = 0.020).

### Global- and nodal-level network indices

**FIGURE 2:**
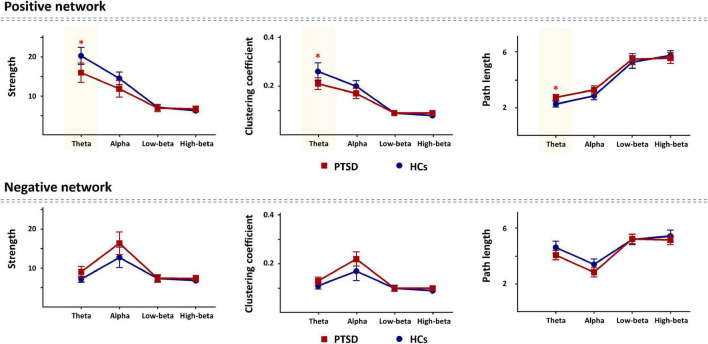
Comparison of the global-level network indices (strength, clustering coefficient, and path length) in all frequency bands for each positive and negative network between the post-traumatic stress disorder (PTSD) patients and healthy controls (HCs). PTSD, post-traumatic stress disorder; HCs, healthy controls. *p < 0.01.

For global-level network indices, the significant differences between the PTSD patients and HCs were observed when using only the task-specific positive network indices, but there was no significant difference in the task-specific negative network indices between the two groups. Figure 2 represents all statistical results of global-level network indices for both positive and negative networks, regardless of their significances. For the global-level network, the PTSD patients showed reduced strength and clustering coefficient, but increased path length in theta frequency band (*p* < 0.01), compared with those of the HCs. Tables 2, 3 summarize all statistical results (e.g., mean and standard deviation, *p*-value, *t*-value, degree of freedom, and effect size) for the task-specific both positive and negative networks at global-level, regardless of their statistical significances, respectively.

**TABLE 2:** All statistic results of global-level network indices for positive network between two groups.

	PTSD	HCs	*p*-value	*t*-values	DF	Effect size
**Theta**						
Strength	15.98 ± 7.57	20.30 ± 8.33	0.009*	–2.56	77.35	0.55
Clustering coefficient	0.21 ± 0.09	0.26 ± 0.11	0.008*	–2.55	76.23	0.55
Path length	2.75 ± 0.81	2.27 ± 0.65	0.003*	3.15	89.30	0.64
**Alpha**						
Strength	11.85 ± 6.62	14.51 ± 6.13	0.051	–1.98	85.27	0.41
Clustering coefficient	0.17 ± 0.08	0.20 ± 0.07	0.088	–1.72	87.24	0.36
Path length	3.29 ± 1.10	2.86 ± 0.87	0.038	2.11	89.44	0.42
**Low-beta**						
Strength	6.93 ± 2.89	7.11 ± 2.61	0.753	–0.32	86.33	0.07
Clustering coefficient	0.09 ± 0.04	0.09 ± 0.03	0.891	–0.14	86.84	0.02
Path length	5.49 ± 1.49	5.28 ± 1.39	0.490	0.69	85.10	0.15
**High-beta**						
Strength	6.67 ± 2.40	6.23 ± 1.91	0.327	0.99	89.37	0.20
Clustering coefficient	0.09 ± 0.03	0.08 ± 0.02	0.214	1.25	89.99	0.25
Path length	5.57 ± 1.49	5.75 ± 1.08	0.517	–0.65	89.98	0.13

*p < 0.01.

DF, degree of freedom.

**TABLE 3:** All statistic results of global-level network indices for negative network between two groups.

	PTSD	HCs	*p*-value	*t*-values	DF	Effect size
**Theta**						
Strength	9.04 ± 4.42	7.19 ± 2.93	0.019	2.41	89.14	0.48
Clustering coefficient	0.13 ± 0.06	0.11 ± 0.04	0.065	1.89	89.97	0.38
Path length	4.06 ± 1.28	4.61 ± 1.39	0.055	–1.95	78.24	0.42
**Alpha**						
Strength	16.38 ± 8.93	12.74 ± 3.02	0.075	1.81	76.64	0.39
Clustering coefficient	0.22 ± 0.11	0.17 ± 0.12	0.083	1.76	76.75	0.38
Path length	2.83 ± 1.23	3.40 ± 1.17	0.028	–2.24	84.14	0.47
**Low-beta**						
Strength	7.40 ± 2.98	7.35 ± 3.02	0.937	0.08	81.53	0.02
Clustering coefficient	0.10 ± 0.04	0.10 ± 0.04	0.770	0.29	80.98	0.06
Path length	5.22 ± 1.31	5.18 ± 1.14	0.889	0.14	87.41	0.03
**High-beta**						
Strength	7.37 ± 2.46	6.83 ± 2.32	0.284	1.08	84.52	0.23
Clustering coefficient	0.10 ± 0.03	0.09 ± 0.03	0.133	1.52	85.31	0.32
Path length	5.15 ± 1.27	5.42 ± 1.34	0.335	–0.97	79.48	0.21

DF, degree of freedom.

**FIGURE 3:**
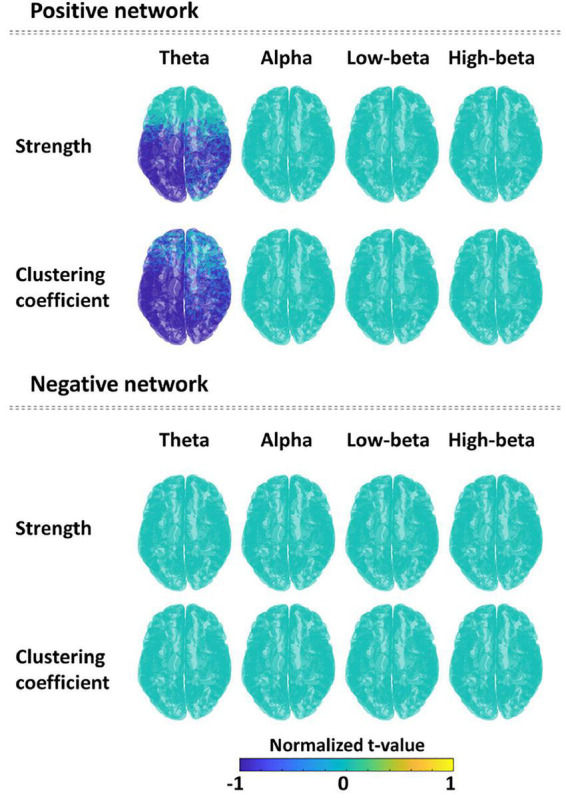
Comparison of the nodal network indices (strength and clustering coefficient) between the post-traumatic stress disorder (PTSD) patients and healthy controls (HCs) in all frequency bands for each positive and negative network. Spatial distribution is described using normalized *t*-values (range: –1 to 1) between two groups for each nodal network index and each frequency band. Blue color means the significantly reduced nodal network values in PTSD patients compared to HCs.

Significant differences of nodal-level network indices between the PTSD patients and HCs were revealed only in the task-specific positive network. For the positive network, PTSD patients showed the significantly reduced nodal strengths (cluster-*p* = 0.018) and nodal clustering coefficients (cluster-*p* = 0.005), predominantly located in parieto-temporo-occipital regions, in theta frequency band compared to those of HCs. On the other hand, there was no significant difference between PTSD patients and HCs in task-specific negative network. Figure 3 depicts the statistical results of nodal-level network indices based on *t*-values. Supplementary Table 2 reports all statistical results (e.g., mean and standard deviation, *t*-value, and effect size) of nodal-level network indices between the two groups.

### Relationships between the network indices and psychiatric symptom scores

**FIGURE 4:**
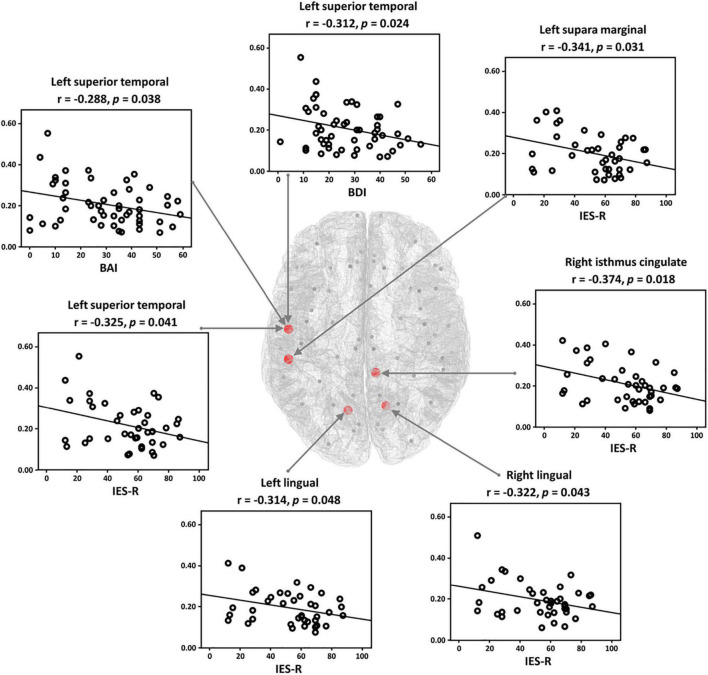
Relationships between the symptom scores [Impact Event Scale-Revised (IES-R), Back’s Depression Inventory (BDI), and Back’s Anxiety Inventory (BAI)] and nodal clustering coefficient in theta frequency band in the post-traumatic stress disorder (PTSD) patients. The clustering coefficient of each patient has significantly negative correlations with symptom scores.

Correlation analysis was conducted for all network indices of global-and nodal-level for both positive and negative networks. Note that the correlation analysis was performed for both PTSD patients and HCs independently, but significant relationships were observed only in PTSD patients. In the case of the global-level, there was no significant relationship between the symptom scores and network indices. For the nodal-level, significant relationships were revealed only in the positive network. In the theta frequency band, significantly negative correlations were observed between the nodal clustering coefficients of five brain regions (left superior temporal, left supari marginal, left and right lingual, and right isthmus cingulate) and IES-R. Moreover, the clustering coefficients of left superior temporal were correlated with depression (BDI) and anxiety symptom scores (BAI), respectively (Figure 4).

## Discussion

In this study, we investigated the altered cortical network characteristics related to the declined cognitive function of PTSD patients by using EEG recorded during performing a simple auditory cognitive task. The PTSD patients showed diminished spectral powers around P300 periods and disrupted network characteristics (reduced strength and clustering coefficient, and prolonged path length) in theta frequency band, compared with those of the HCs. Moreover, the PTSD patients displayed disrupted network indices in specific brain regions for task-specific positive networks. The nodal strengths and nodal clustering coefficients of PTSD patients were significantly reduced in theta frequency band compared with those of the HCs. We also observed the significant relationships between the nodal clustering coefficients of the task-specific positive network and psychiatric symptom scores (IES-R, BDI, and BAI) in theta band. The distinct brain network characteristics associated with the psychiatric symptoms of PTSD patients can provide neurophysiologically meaningful information to improve the understanding of the neuropathology related to PTSD.

Interestingly, in this study, distinct disease-specific network patterns were revealed when using only task-specific enhanced connectomes. It is obvious that, compared with the baseline, significantly enhanced functional connections were observed during cognitive processing, but no diminished connection was detected (42, 43). Therefore, most previous studies, which have investigated the neuronal characteristics of psychiatric disorders, have focused on investigating the enhanced connectomes during cognitive processing compared with the baseline rather than investigating the diminished connectomes (44, 45). For example, schizophrenia patients showed significantly fewer enhanced connections than healthy individuals when processing cognitive tasks (44). Additionally, these patients are inefficient in creating the enhanced connections needed to appropriately process cognitive tasks, leading to the impaired cognitive function. Therefore, our study is in line with the results from previous studies in that the significant results were only observed when using the enhanced (positive) connectomes. Moreover, since there has been no study investigating the disrupted enhanced connections in PTSD patients during processing cognitive tasks, our study provides a better understanding of the pathology of the impaired cognitive function in PTSD patients by investigating the functional network indices based on task-specific increased connectomes.

To date, most studies have investigated the impaired cognitive function in PTSD patients by using simple ERP characteristics, such as reduced amplitudes and prolonged latencies, which can provide neurophysiologically meaningful information (e.g., how fewer brain resources are allocated to cognitive processing or how slow processing speed is in PTSD patients) (46). However, such fragmentary information obtained from simple time-series ERP traits would be insufficient to fully comprehend the neuronal mechanisms involved in the cognitive function occurring during complex systematic interactions based on the network structure (19). Moreover, simple ERP studies tend to overlook the frequency-domain information that can represent the impaired cognition-related psychopathology of PTSD. For example, it has been well documented that PTSD patients show altered theta patterns during the resting-state even though neurophysiological findings are still controversial [enhanced (47) vs. diminished (48, 49) theta power]. Nevertheless, a reliable consensus has been reached that the theta oscillation is closely related to cognitive functions, such as attention and memory processing (50–53). Therefore, insightful information can be obtained by investigating the unique network characteristics along with frequency-domain information to have a better understanding of the impaired cognitive mechanism in PTSD patients. Indeed, in this study, the PTSD patients showed abnormal theta frequency information in terms of both total power (reduced spectral power) and network indices (weaker strength, lower clustering coefficient, and prolonged path length) covering the whole-brain during simple attention processing, as compared with those of the HCs. In line with previous studies, PTSD patients cannot allocate enough brain resource to boost spectral power of theta frequency band during task as compared to HCs. Moreover, the weaker strength and lower clustering coefficient indicate that the connections between specific brain regions are loose, and the prolonged path length indicates that the processing speed is slower in PTSD patients than in HCs. Consequently, this impairment in brain networks hinder the appropriate cognitive processing, leading to the impaired cognitive function in PTSD patients.

In terms of brain regional view, the PTSD patients showed significantly reduced nodal clustering coefficients of task-specific positive network in theta frequency band, dominantly located in parieto-temporo-occipital regions such as superior temporal, parahippocampal gyrus, lingual gyrus, etc., compared with those of the HCs. Particularly, temporal regions are generally involved in cognition-related information processing, especially memory processing (54). In addition, the parahippocampal gyrus is known to be a bridge region in the information-processing pathway from the temporal areas to the hippocampus regarding the memory process (encoding and retrieval) (51). Accordingly, the diminished theta clustering coefficients of the PTSD patients in those of brain regions implied that both the bridge of the memory pathway and the direct memory related regions do not properly work in PTSD patients, finally resulting in the impaired cognitive function. Furthermore, negative correlations were revealed between the nodal clustering coefficients in several regions (isthmus cingulate, lingual, superior temporal, and supra marginal) in theta frequency band and the symptom scores (IES-R for the degrees of stress from the trauma-related events, BDI for depression symptoms, and BAI for anxiety), respectively. The correlation results imply that nodal clustering coefficients of the brain regions get lower as the degree of psychiatric symptoms increases. These results indicate that parieto-temporo-occipital network is more altered in severe PTSD patients as compared to those of relatively intact patients, leading to poorer attention processing of severe PTSD patients.

There are three main limitations to the present study. First, since all recruited PTSD patients were on medication, we could not control the compounding effects from medications. Second, even though we controlled for other psychiatric illness, we did not control for comorbid depression. Third, although demographic data were statistically matched between two groups, the number of participants was unbalanced between two participant groups.

## Conclusion

Although the impaired cognitive function is accounted as an important symptom in PTSD patients, the related brain networks have not been investigated before. We first attempted to explore the brain networks related to impaired cognitive function in PTSD patients by using a simple auditory oddball task and finally identified the modified brain-network characteristics in PTSD patients compared with HCs. PTSD patients showed disrupted whole-brain network indices (lower strength and clustering coefficient and prolonged path length) in theta frequency band, compared with those of the HCs. Moreover, diminished nodal clustering coefficients in theta band were discovered in parieto-temporo-occipital regions, and decreased nodal clustering coefficients of the parieto-temporo-occipital regions were found to have significant relationships with the psychiatric symptom scores. The presented results enhance the understanding of the impaired cognitive pathology in PTSD patients by revealing the altered brain-networks characteristics and thereby present the network indices as promising biomarkers of PTSD.

## Data availability statement

The raw data supporting the conclusions of this article will be made available by the authors, without undue reservation.

## Ethics statement

The study involving human participants was reviewed and approved by the Institutional Review Board of Inje University Ilsan Paik Hospital (2015-07-025). The patients/participants provided their written informed consent to participate in this study.

## Author contributions

MS, H-JH, and S-HL: conception/design of the work, interpretation of data, and writing (original draft preparation and editing). MS and S-HL: data acquisition and analysis. All authors read and contributed to the final version of the manuscript.

## Footnotes

1
http://www.brain-connectivity-toolbox.net

## References

[1] Cassiday KL Mcnally RJ Zeitlin SB. Cognitive processing of trauma cues in rape victims with post-traumatic stress disorder. *Cognit Ther Res.* (1992) 16:283–95. 10.1037//0022-006x.67.5.705

[2] Francati V Vermetten E Bremner J. Functional neuroimaging studies in posttraumatic stress disorder: Review of current methods and findings. *Depress Anxiety.* (2007) 24:202–18. 16960853 10.1002/da.20208 PMC3233765

[3] Hughes KC Shin LM. Functional neuroimaging studies of post-traumatic stress disorder. *Expert Rev Neurother.* (2011) 11:275–85. 21306214 10.1586/ern.10.198 PMC3142267

[4] Bomyea J Johnson A Lang AJ. Information processing in PTSD: Evidence for biased attentional, interpretation, and memory processes. *Psychopathol Rev.* (2017) 4:218–43. 10.3389/fnint.2012.00089 23087624 PMC3466464

[5] Nejati V Salehinejad MA Sabayee A. Impaired working memory updating affects memory for emotional and non-emotional materials the same way: Evidence from post-traumatic stress disorder (PTSD). *Cogn Process.* (2018) 19:53–62. 10.1007/s10339-017-0837-2 28929242

[6] Shin LM Rauch SL Pitman RK. Amygdala, medial prefrontal cortex, and hippocampal function in PTSD. *Ann N Y Acad Sci.* (2006) 1071:67–79. 16891563 10.1196/annals.1364.007

[7] Yun J-Y Jin MJ Kim S Lee S-H. Stress-related cognitive style is related to volumetric change of the hippocampus and FK506 binding protein 5 polymorphism in post-traumatic stress disorder. *Psychol Med.* (2020) 52:1243–54. 10.1017/S0033291720002949 32892762

[8] Bryant RA Felmingham KL Kemp AH Barton M Peduto AS Rennie C Neural networks of information processing in posttraumatic stress disorder: A functional magnetic resonance imaging study. *Biol Psychiatry.* (2005) 58:111–8. 16038681 10.1016/j.biopsych.2005.03.021

[9] Morey RA Dolcos F Petty CM Cooper DA Hayes JP Labar KS The role of trauma-related distractors on neural systems for working memory and emotion processing in posttraumatic stress disorder. *J Psychiatr Res.* (2009) 43:809–17. 10.1016/j.jpsychires.2008.10.014 19091328 PMC2684984

[10] Aupperle RL Melrose AJ Stein MB Paulus MP. Executive function and PTSD: Disengaging from trauma. *Neuropharmacology.* (2012) 62:686–94. 21349277 10.1016/j.neuropharm.2011.02.008 PMC4719148

[11] D’Esposito M Postle BR Rypma B. Prefrontal cortical contributions to working memory: Evidence from event-related fMRI studies. Executive control and the frontal lobe. *Curr Issues.* (2000) 133:3–11. 10.1006/nimg.2001.0936 10933205

[12] West HV Burgess GC Dust J Kandala S Barch DM. Amygdala activation in cognitive task fMRI varies with individual differences in cognitive traits. *Cogn Affect Behav Neurosci.* (2021) 21:254–64. 10.3758/s13415-021-00863-3 33683660 PMC8480985

[13] Polich J. Updating P300: An integrative theory of P3a and P3b. *Clin. Neurophysiol.* (2007) 118:2128–48. 10.1016/j.clinph.2007.04.019 17573239 PMC2715154

[14] Srinivasan N. Cognitive neuroscience of creativity: EEG based approaches. *Methods.* (2007) 42:109–16. 17434421 10.1016/j.ymeth.2006.12.008

[15] Beres AM. Time is of the essence: A review of electroencephalography (EEG) and event-related brain potentials (ERPs) in language research. *Appl Psychophysiol Biofeedback.* (2017) 42:247–55. 10.1007/s10484-017-9371-3 28698970 PMC5693972

[16] Min D Kwon A Kim Y Jin MJ Kim Y-W Jeon H Clinical implication of altered inhibitory response in patients with post-traumatic stress disorder: Electrophysiological evidence from a Go/Nogo task. *Brain Topogr.* (2020) 33:208–20. 10.1007/s10548-020-00754-9 32034577

[17] Felmingham KL Bryant RA Kendall C Gordon E. Event-related potential dysfunction in posttraumatic stress disorder: The role of numbing. *Psychiatry Res.* (2002) 109:171–9. 10.1016/s0165-1781(02)00003-3 11927142

[18] Araki T Kasai K Yamasue H Kato N Kudo N Ohtani T Association between lower P300 amplitude and smaller anterior cingulate cortex volume in patients with posttraumatic stress disorder: A study of victims of Tokyo subway sarin attack. *Neuroimage.* (2005) 25:43–50. 10.1016/j.neuroimage.2004.11.039 15734342

[19] Braun U Schäfer A Walter H Erk S Romanczuk-Seiferth N Haddad L Dynamic reconfiguration of frontal brain networks during executive cognition in humans. *Proc Natl Acad Sci USA.* (2015) 112:11678–83. 26324898 10.1073/pnas.1422487112 PMC4577153

[20] Shim M Kim D-W Lee S-H Im C-H. Disruptions in small-world cortical functional connectivity network during an auditory oddball paradigm task in patients with schizophrenia. *Schizophr Res.* (2014) 156:197–203. 10.1016/j.schres.2014.04.012 24819192

[21] Rommel A-S Kitsune GL Michelini G Hosang GM Asherson P Mcloughlin G Commonalities in EEG spectral power abnormalities between women with ADHD and women with bipolar disorder during rest and cognitive performance. *Brain Topogr.* (2016) 29:856–66. 10.1007/s10548-016-0508-0 27464584 PMC5054048

[22] Kazemi R Rostami R Khomami S Baghdadi G Rezaei M Hata M Bilateral transcranial magnetic stimulation on DLPFC changes resting state networks and cognitive function in patients with bipolar depression. *Front Hum Neurosci.* (2018) 12:356. 10.3389/fnhum.2018.00356 30233346 PMC6135217

[23] Beck AT Steer RA. *Manual for the Beck anxiety inventory.* San Antonio, TX: Psychological Corporation (1990).

[24] Yook S Kim Z. A clinical study on the Korean version of Beck anxiety inventory: Comparative study of patient and non-patient. *Korean J Clin Psychol.* (1997) 16:185–97.

[25] Beck AT Steer RA Brown GK. *Beck depression inventory-II.* San Antonio, TX: Psychological Corporation (1996). p. 78204–72498.

[26] Cha JM Kim JE Kim MA Shim B Cha MJ Lee JJ Five months follow-up study of school-based crisis intervention for Korean high school students who experienced a peer suicide. *J Korean Med Sci.* (2018) 33:e192. 10.3346/jkms.2018.33.e192 29983694 PMC6033103

[27] Weiss DS. The impact of event scale: Revised. In: Wilson JP Tang CS editors. *Cross-cultural assessment of psychological trauma and PTSD.* (Boston, MA: Springer) (2007). p. 219–38. 10.3390/jpm12050681

[28] Lim H-K Woo J-M Kim T-S Kim T-H Choi K-S Chung S-K Reliability and validity of the Korean version of the impact of event scale-revised. *Compr Psychiatry.* (2009) 50:385–90. 19486738 10.1016/j.comppsych.2008.09.011

[29] Sohn JH Lim J Lee JS Kim K Lim S Byeon N Prevalence of possible depression and post-traumatic stress disorder among community dwelling adult refugees and refugee applicants in South Korea. *J Korean Med Sci.* (2019) 34:e97. 10.3346/jkms.2019.34.e97 30914907 PMC6427049

[30] Semlitsch HV Anderer P Schuster P Presslich O. A solution for reliable and valid reduction of ocular artifacts, applied to the P300 ERP. *Psychophysiology.* (1986) 23:695–703. 10.1111/j.1469-8986.1986.tb00696.x 3823345

[31] Kim J-Y Son J-B Leem H-S Lee S-H. Psychophysiological alteration after virtual reality experiences using smartphone-assisted head mount displays: An EEG-based source localization study. *Appl Sci.* (2019) 9:2501.

[32] Gramfort A Papadopoulo T Olivi E Clerc M. OpenMEEG: Opensource software for quasistatic bioelectromagnetics. *Biomed Eng Online.* (2010) 9:45. 10.1186/1475-925X-9-45 20819204 PMC2949879

[33] Tadel F Baillet S Mosher JC Pantazis D Leahy RM. Brainstorm: A user-friendly application for MEG/EEG analysis. *Comput Intell Neurosci.* (2011) 2011:879716. 10.1155/2011/879716 21584256 PMC3090754

[34] Dimitriadis SI López ME Bruña R Cuesta P Marcos A Maestú F How to build a functional connectomic biomarker for mild cognitive impairment from source reconstructed MEG resting-state activity: The combination of ROI representation and connectivity estimator matters. *Front Neurosci.* (2018) 12:306. 10.3389/fnins.2018.00306 29910704 PMC5992286

[35] Fraschini M Demuru M Crobe A Marrosu F Stam CJ Hillebrand A. The effect of epoch length on estimated EEG functional connectivity and brain network organisation. *J Neural Eng.* (2016) 13:036015. 10.1088/1741-2560/13/3/036015 27137952

[36] Tóth B Urbán G Haden GP Márk M Török M Stam CJ Large-scale network organization of EEG functional connectivity in newborn infants. *Hum Brain Mapp.* (2017) 38:4019–33. 28488308 10.1002/hbm.23645 PMC6867159

[37] Shim M Im C-H Lee S-H. Disrupted cortical brain network in post-traumatic stress disorder patients: A resting-state electroencephalographic study. *Transl Psychiatry.* (2017) 7:e1231. 10.1038/tp.2017.200 28895942 PMC5639244

[38] Rubinov M Sporns O. Complex network measures of brain connectivity: Uses and interpretations. *Neuroimage.* (2010) 52:1059–69. 19819337 10.1016/j.neuroimage.2009.10.003

[39] Maris E Oostenveld R. Nonparametric statistical testing of EEG-and MEG-data. *J Neurosci Methods.* (2007) 164:177–90. 17517438 10.1016/j.jneumeth.2007.03.024

[40] Groppe DM Urbach TP Kutas M. Mass univariate analysis of event-related brain potentials/fields I: A critical tutorial review. *Psychophysiology.* (2011) 48:1711–25. 10.1111/j.1469-8986.2011.01273.x 21895683 PMC4060794

[41] Rajkumar R Farrher E Mauler J Sripad P Régio Brambilla C Rota Kops E Comparison of EEG microstates with resting state fMRI and FDG-PET measures in the default mode network via simultaneously recorded trimodal (PET/MR/EEG) data. *Hum Brain Mapp.* (2021) 42:4122–33. 10.1002/hbm.24429 30367727 PMC8356993

[42] Choi JW Jung K-Y Kim CH Kim KH. Changes in gamma-and theta-band phase synchronization patterns due to the difficulty of auditory oddball task. *Neurosci Lett.* (2010) 468:156–60. 10.1016/j.neulet.2009.10.088 19883733

[43] Guo M Xu G Wang L Fu L. Functional brain network analysis during auditory oddball task. *Proceedings of the 2016 Asia-Pacific International Symposium on Electromagnetic Compatibility (APEMC).* (Manhattan, NY: IEEE) (2016). p. 1098–100.

[44] Bachiller A Poza J Gómez C Molina V Suazo V Hornero R. A comparative study of event-related coupling patterns during an auditory oddball task in schizophrenia. *J Neural Eng.* (2014) 12:016007. 10.1088/1741-2560/12/1/016007 25474418

[45] Choi JW Jang K-M Jung K-Y Kim M-S Kim KH. Reduced theta-band power and phase synchrony during explicit verbal memory tasks in female, non-clinical individuals with schizotypal traits. *PLoS One.* (2016) 11:e0148272. 10.1371/journal.pone.0148272 26840071 PMC4739585

[46] Whelan R Lonergan R Kiiski H Nolan H Kinsella K Hutchinson M Impaired information processing speed and attention allocation in multiple sclerosis patients versus controls: A high-density EEG study. *J Neurol Sci.* (2010) 293:45–50. 10.1016/j.jns.2010.03.010 20399448

[47] Imperatori C Farina B Quintiliani MI Onofri A Gattinara PC Lepore M Aberrant EEG functional connectivity and EEG power spectra in resting state post-traumatic stress disorder: A sLORETA study. *Biol Psychol.* (2014) 102:10–7. 10.1016/j.biopsycho.2014.07.011 25046862

[48] Franke LM Walker WC Hoke KW Wares JR. Distinction in EEG slow oscillations between chronic mild traumatic brain injury and PTSD. *Int J Psychophysiol.* (2016) 106:21–9. 10.1016/j.ijpsycho.2016.05.010 27238074

[49] Meyers J Mccutcheon VV Pandey AK Kamarajan C Subbie S Chorlian D Early sexual trauma exposure and neural response inhibition in adolescence and young adults: Trajectories of frontal theta oscillations during a go/no-go task. *J Am Acad Child Adolesc Psychiatry.* (2019) 58:242.–255. 10.1016/j.jaac.2018.07.905 30738551 PMC6537865

[50] Buzsáki G. Theta oscillations in the hippocampus. *Neuron.* (2002) 33:325–40. 11832222 10.1016/s0896-6273(02)00586-x

[51] Hasselmo ME. What is the function of hippocampal theta rhythm?–Linking behavioral data to phasic properties of field potential and unit recording data. *Hippocampus.* (2005) 15:936–49. 10.1002/hipo.20116 16158423

[52] Shin J. The interrelationship between movement and cognition: Theta rhythm and the P300 event-related potential. *Hippocampus.* (2011) 21:744–52. 10.1002/hipo.20792 20865727

[53] Wang X Ding M. Relation between P300 and event-related theta-band synchronization: A single-trial analysis. *Clin Neurophysiol.* (2011) 122:916–24. 10.1016/j.clinph.2010.09.011 20943435

[54] Kerr KM Agster KL Furtak SC Burwell RD. Functional neuroanatomy of the parahippocampal region: The lateral and medial entorhinal areas. *Hippocampus.* (2007) 17:697–708. 17607757 10.1002/hipo.20315

