# Supplementary material for: Impaired functional cortical networks in the theta frequency band of patients with post-traumatic stress disorder during auditory-cognitive processing
Source: Front Psychiatry. 2022 Aug 11;13:811766. doi: 10.3389/fpsyt.2022.811766 (PMC9403077; doi:10.3389/fpsyt.2022.811766)
Supplement: Supplementary file 1 [file Table_1.DOCX]

**Supplementary Table 1.** Regions of interest (ROI) based on the Desikan-Killian atlas. A ROI pair represents same brain area of each hemisphere (left and right), and each ROI pair is assigned to the same lobe, e.g., ROI 1 and 2 represent left Bankssts and right Bankssts of the temporal lobe, respectively.

| **Number** | **Name** |  | **Number** | **Name** | **Lobe** |
| --- | --- | --- | --- | --- | --- |
| 1 | Bankssts L |  | 2 | Bankssts R | Temporal |
| 3 | Caudal anterior cingulate L |  | 4 | Caudal anterior cingulate R | Frontal |
| 5 | Caudal middle frontal L |  | 6 | Caudal middle frontal R | Frontal |
| 7 | Cuneus L |  | 8 | Cuneus R | Occipital |
| 9 | Entorhinal L |  | 10 | Entorhinal R | Temporal |
| 11 | Frontal pole L |  | 12 | Frontal pole R | Frontal |
| 13 | Fusiform L |  | 14 | Fusiform R | Temporal |
| 15 | Inferior parietal L |  | 16 | Inferior parietal R | Parietal |
| 17 | Inferior temporal L |  | 18 | Inferior temporal R | Temporal |
| 19 | Insula L |  | 20 | Insula R | Temporal |
| 21 | Isthmus cingulate L |  | 22 | Isthmus cingulate R | Parietal |
| 23 | Lateral occipital L |  | 24 | Lateral occipital R | Occipital |
| 25 | Lateral orbitofrontal L |  | 26 | Lateral orbitofrontal R | Frontal |
| 27 | Lingual L |  | 28 | Lingual R | Occipital |
| 29 | Medial orbitofrontal L |  | 30 | Medial orbitofrontal R | Frontal |
| 31 | Middle temporal L |  | 32 | Middle temporal R | Temporal |
| 33 | Paracentral L |  | 34 | Paracentral R | Frontal |
| 35 | Para hippocampal L |  | 36 | Para hippocampal R | Temporal |
| 37 | Pars opercularis L |  | 38 | Pars opercularis R | Frontal |
| 39 | Pars orbitalis L |  | 40 | Pars orbitalis R | Frontal |
| 41 | Pars triangularis L |  | 42 | Pars triangularis R | Frontal |
| 43 | Pericalcarine L |  | 44 | Pericalcarine R | Occipital |
| 45 | Post central L |  | 46 | Postcentral R | Parietal |
| 47 | Posterior cingulate L |  | 48 | Posterior cingulate R | Parietal |
| 49 | Precentral L |  | 50 | Precentral R | Frontal |
| 51 | Precuneus L |  | 52 | Precuneus R | Parietal |
| 53 | Rostral anterior cingulate L |  | 54 | Rostral anterior cingulate R | Frontal |
| 55 | Rostral middle frontal L |  | 56 | Rostral middle frontal R | Frontal |
| 57 | Superior frontal L |  | 58 | Superior frontal R | Frontal |
| 59 | Superior parietal L |  | 60 | Superior parietal R | Parietal |
| 61 | Superior temporal L |  | 62 | Superior temporal R | Temporal |
| 63 | Supramarginal L |  | 64 | Supramarginal R | Parietal |
| 65 | Temporal pole L |  | 66 | Temporal pole R | Temporal |
| 67 | Transverse temporal L |  | 68 | Transverse temporal R | Temporal |
