# Supplementary material for: Impaired functional cortical networks in the theta frequency band of patients with post-traumatic stress disorder during auditory-cognitive processing
Source: Front Psychiatry. 2022 Aug 11;13:811766. doi: 10.3389/fpsyt.2022.811766 (PMC9403077; doi:10.3389/fpsyt.2022.811766)
Supplement: Supplementary file 2 [file Table_2.DOCX]

**Supplementary Table 2.** Regions of Interests information and statistical results showing significant differences in nodal-level network indices between the two groups of post-traumatic stress disorder (PTSD) patients and healthy controls (HCs) for positive networks. However, there was no significant difference between the two groups for negative network.

**Task-specific positive network**

|  | **PTSD** | **HCs** | ***t*-value** | **Effect size** |
| --- | --- | --- | --- | --- |
| **Strengths (cluster-*p* = 0.005, degree of freedom = 90)** | | | | |
| Cuneus L | 12.51 ± 7.98 | 17.41 ± 11.80 | -2.38 | 0.50 |
| Fusiform L | 12.96 ± 6.99 | 18.70 ± 10.69 | -3.11 | 0.66 |
| Inferior parietal L | 12.37 ± 8.12 | 18.49 ± 13.80 | -2.66 | 0.56 |
| Inferior temporal L | 12.85 ± 7.64 | 17.64 ± 11.14 | -2.45 | 0.52 |
| Isthmus cingulate L | 15.20 ± 11.77 | 21.00 ± 13.45 | -2.20 | 0.46 |
| Lateral occipital L | 12.84 ± 8.14 | 17.32 ± 10.67 | -2.28 | 0.48 |
| Lingual L | 14.55 ± 9.15 | 19.35 ± 12.97 | -2.08 | 0.44 |
| Middle temporal L | 14.57 ± 8.86 | 19.69 ± 13.63 | -2.18 | 0.46 |
| Para hippocampal L | 13.43 ± 8.64 | 21.41 ± 12.78 | -3.57 | 0.75 |
| Pericalcarine L | 12.81 ± 6.97 | 19.34 ± 14.29 | -2.89 | 0.61 |
| Post central L | 15.09 ± 11.09 | 22.83 ± 12.78 | -3.10 | 0.65 |
| Posterior cingulate L | 17.14 ± 11.25 | 26.53 ± 19.58 | -2.90 | 0.61 |
| Precentral L | 16.54 ± 12.82 | 23.28 ± 12.77 | -2.50 | 0.53 |
| Precuneus L | 13.08 ± 9.59 | 20.47 ± 15.51 | -2.82 | 0.59 |
| Superior parietal L | 14.23 ± 8.29 | 20.64 ± 15.05 | -2.61 | 0.55 |
| Superior temporal L | 15.00 ± 10.18 | 20.57 ± 14.31 | -2.18 | 0.46 |
| Supra marginal L | 13.97 ± 10.17 | 21.99 ± 14.70 | -3.09 | 0.65 |
| Transverse temporal L | 14.27 ± 9.37 | 19.21 ± 12.50 | -2.17 | 0.46 |
| Cuneus R | 12.29 ± 9.53 | 17.58 ± 13.93 | -2.16 | 0.46 |
| Isthmus cingulate R | 15.56 ± 11.37 | 23.10 ± 16.68 | -2.58 | 0.54 |
| Lingual R | 14.45 ± 10.19 | 23.04 ± 17.80 | -2.93 | 0.62 |
| Middle temporal R | 13.63 ± 8.94 | 17.95 ± 11.12 | -2.06 | 0.44 |
| Pericalcarine R | 13.67 ± 10.11 | 21.04 ± 14.14 | -2.92 | 0.62 |
| Postcentral R | 13.86 ± 8.63 | 20.80 ± 12.08 | -3.22 | 0.68 |
| Posterior cingulate R | 17.33 ± 10.93 | 24.55 ± 18.27 | -2.36 | 0.50 |
| Precuneus R | 14.03 ± 10.42 | 20.66 ± 15.75 | -2.43 | 0.51 |
| Superior parietal R | 13.88 ± 9.39 | 19.74 ± 16.39 | -2.17 | 0.46 |
| Superior temporal R | 13.69 ± 10.20 | 18.69 ± 10.48 | -2.30 | 0.48 |
| Supra marginal R | 13.74 ± 8.52 | 19.47 ± 10.09 | -2.95 | 0.62 |
| Transverse temporal R | 13.26 ± 10.13 | 18.64 ± 11.33 | -2.39 | 0.50 |
|  | | | | |
| **Clustering coefficients (cluster-*p* = 0.018, degree of freedom = 90)** | | | | |
| Bankssts L | 0.19 ± 0.10 | 0.25 ± 0.12 | -2.81 | 0.59 |
| Caudal middle frontal L | 0.23 ± 0.13 | 0.29 ± 0.14 | -2.13 | 0.45 |
| Cuneus L | 0.19 ± 0.10 | 0.24 ± 0.11 | -2.39 | 0.51 |
| Entorhinal L | 0.20 ± 0.09 | 0.26 ± 0.09 | -3.08 | 0.65 |
| Fusiform L | 0.19 ± 0.08 | 0.27 ± 0.12 | -3.72 | 0.79 |
| Inferior parietal L | 0.18 ± 0.11 | 0.24 ± 0.14 | -2.36 | 0.50 |
| Inferior temporal L | 0.20 ± 0.09 | 0.25 ± 0.10 | -2.33 | 0.49 |
| Insula L | 0.22 ± 0.10 | 0.28 ± 0.11 | -2.79 | 0.59 |
| Isthmus cingulate L | 0.22 ± 0.12 | 0.29 ± 0.13 | -2.78 | 0.59 |
| Lateral occipital L | 0.18 ± 0.08 | 0.25 ± 0.12 | -2.96 | 0.62 |
| Lateral orbitofrontal L | 0.23 ± 0.13 | 0.28 ± 0.11 | -2.04 | 0.43 |
| Lingual L | 0.20 ± 0.08 | 0.26 ± 0.11 | -3.05 | 0.64 |
| Middle temporal L | 0.21 ± 0.10 | 0.26 ± 0.12 | -2.43 | 0.51 |
| Paracentral L | 0.22 ± 0.11 | 0.28 ± 0.11 | -2.37 | 0.50 |
| Para hippocampal L | 0.21 ± 0.08 | 0.28 ± 0.12 | -3.58 | 0.76 |
| Pars orbitalis L | 0.22 ± 0.11 | 0.27 ± 0.12 | -2.32 | 0.49 |
| Pericalcarine L | 0.19 ± 0.08 | 0.25 ± 0.12 | -2.94 | 0.62 |
| Postcentral L | 0.20 ± 0.12 | 0.28 ± 0.13 | -3.08 | 0.65 |
| Posterior cingulate L | 0.22 ± 0.11 | 0.30 ± 0.15 | -3.01 | 0.63 |
| Precentral L | 0.22 ± 0.12 | 0.29 ± 0.13 | -2.78 | 0.59 |
| Precuneus L | 0.20 ± 0.11 | 0.27 ± 0.14 | -2.58 | 0.54 |
| Superior frontal L | 0.24 ± 0.13 | 0.33 ± 0.16 | -2.97 | 0.63 |
| Superior parietal L | 0.20 ± 0.10 | 0.27 ± 0.16 | -2.39 | 0.50 |
| Superior temporal L | 0.21 ± 0.10 | 0.28 ± 0.13 | -2.91 | 0.61 |
| Supra marginal L | 0.20 ± 0.11 | 0.27 ± 0.14 | -2.79 | 0.59 |
| Temporal pole L | 0.21 ± 0.12 | 0.28 ± 0.14 | -2.56 | 0.54 |
| Transverse temporal L | 0.21 ± 0.10 | 0.27 ± 0.13 | -2.53 | 0.53 |
| Bankssts R | 0.19 ± 0.10 | 0.24 ± 0.10 | -2.10 | 0.44 |
| Caudal anterior cingulate R | 0.23 ± 0.12 | 0.30 ± 0.13 | -2.33 | 0.49 |
| Cuneus R | 0.19 ± 0.10 | 0.24 ± 0.13 | -2.21 | 0.47 |
| Inferior parietal R | 0.18 ± 0.10 | 0.23 ± 0.09 | -2.24 | 0.47 |
| Isthmus cingulate R | 0.22 ± 0.12 | 0.29 ± 0.13 | -2.60 | 0.55 |
| Lateral occipital R | 0.19 ± 0.09 | 0.23 ± 0.10 | -2.10 | 0.44 |
| Lingual R | 0.20 ± 0.10 | 0.27 ± 0.13 | -2.83 | 0.60 |
| Paracentral R | 0.23 ± 0.11 | 0.28 ± 0.13 | -2.05 | 0.43 |
| Pericalcarine R | 0.20 ± 0.10 | 0.25 ± 0.13 | -2.32 | 0.49 |
| Postcentral R | 0.19 ± 0.09 | 0.26 ± 0.13 | -3.06 | 0.65 |
| Posterior cingulate R | 0.22 ± 0.11 | 0.30 ± 0.15 | -2.83 | 0.60 |
| Precentral R | 0.21 ± 0.10 | 0.26 ± 0.14 | -2.06 | 0.43 |
| Precuneus R | 0.20 ± 0.11 | 0.27 ± 0.14 | -2.59 | 0.55 |
| Superior frontal R | 0.24 ± 0.13 | 0.30 ± 0.16 | -2.00 | 0.42 |
| Superior parietal R | 0.19 ± 0.10 | 0.25 ± 0.14 | -2.32 | 0.49 |
| Superior temporal R | 0.19 ± 0.10 | 0.24 ± 0.10 | -2.09 | 0.44 |
| Supra marginal R | 0.20 ± 0.11 | 0.24 ± 0.10 | -2.07 | 0.44 |
| Transverse temporal R | 0.20 ± 0.10 | 0.24 ± 0.11 | -2.05 | 0.43 |
